# Cannabidiol Inhibits Tau Aggregation In Vitro

**DOI:** 10.3390/cells10123521

**Published:** 2021-12-13

**Authors:** Soha Alali, Gholamhossein Riazi, Mohammad Reza Ashrafi-Kooshk, Sogol Meknatkhah, Shahin Ahmadian, Mohammad Hooshyari Ardakani, Baharak Hosseinkhani

**Affiliations:** 1 Laboratory of Neuro-Organic Chemistry, Institute of Biochemistry and Biophysics (IBB), University of Tehran, Tehran 1417614335, Iran; mrashrafi@ut.ac.ir (M.R.A.-K.); sogolmeknatkhah@ut.ac.ir (S.M.); 2 Department of Biochemistry, Institute of Biochemistry and Biophysics, University of Tehran, Tehran 1417614335, Iran; sh.ahmadian@ut.ac.ir; 3 Department of Phytochemistry, Medicinal Plants and Drugs Research Institute, Shahid Beheshti University, G.C., Evin, Tehran 1983969411, Iran; M.hoosh1991@gmail.com; 4 Biomedical Research Institute (BIOMED), Hasselt University, Martelarenlaan 42, 3500 Hasselt, Belgium; baharak.hosseinkhani@uhasselt.be

**Keywords:** Alzheimer’s disease, tau protein, amyloid fibrils, cannabidiol

## Abstract

A hallmark of Alzheimer’s disease (AD) is the accumulation of tau protein in the brain. Compelling evidence indicates that the presence of tau aggregates causes irreversible neuronal destruction, eventually leading to synaptic loss. So far, the inhibition of tau aggregation has been recognized as one of the most effective therapeutic strategies. Cannabidiol (CBD), a major component found in *Cannabis sativa* L., has antioxidant activities as well as numerous neuroprotective features. Therefore, we hypothesize that CBD may serve as a potent substance to hamper tau aggregation in AD. In this study, we aim to investigate the CBD effect on the aggregation of recombinant human tau protein 1N/4R isoform using biochemical methods in vitro and in silico. Using Thioflavin T (ThT) assay, circular dichroism (CD), and atomic force microscopy (AFM), we demonstrated that CBD can suppress tau fibrils formation. Moreover, by quenching assay, docking, and job’s plot, we further demonstrated that one molecule of CBD interacts with one molecule of tau protein through a spontaneous binding. Experiments performed by quenching assay, docking, and Thioflavin T assay further established that the main forces are hydrogen Van der Waals and some non-negligible hydrophobic forces, affecting the lag phase of tau protein kinetics. Taken together, this study provides new insights about a natural substance, CBD, for tau therapy which may offer new hope for the treatment of AD.

## 1. Introduction

Alzheimer’s disease (AD) is an irreversible devastating neurodegenerative disease that is mainly diagnosed by memory impairment and cognitive decline due to the slight disruption in the function of the cerebral cortex and hippocampus [1]. Accumulation of β-amyloid (Aβ)-containing extracellular plaques, namely senile plaques (SPs), and tau-containing intracellular neurofibrillary tangles (NFTs) are now known as the two neuropathological hallmarks of AD [2]. In NFTs, polymerization, stability, and organization of microtubules are regulated by the microtubule-associated proteins (MAPs) family. Tau, a member of the MAPs family, is mostly found in the axons of neurons in the central nervous system whose functions are involved in stability and assembly modulation of microtubules, neurite growth, and kinesin-dependent axonal transportation [3,4]. 

Tau is highly hydrophilic and stable at physiological conditions and its aggregation is often induced by polyanions such as heparin, in vitro [5]. Tau protein is a result of translation from a 6-kb mRNA transcript that generates six isoforms. The isoforms of tau protein are differed by the presence of zero, one or two N-terminal inserts (0N, 1N, or 2N, respectively), as well as the presence of either three (3R) or four (4R) microtubule binding repeats (MTBRs) in the C-terminal half of tau [6]. It has been demonstrated that the positive lysine residues in the MTBRs of tau interact with the negative C-terminal of tubulin in a sequence-specific way [7]. In a healthy brain, the ratio of 3R tau isoform is normally equal to 4R tau isoform, while in an AD brain, this ratio is substantially altered more towards the ratio of 2:1 in 4R:3R caused by an increase in the level of 4R tau isoforms or decrease in 3R tau isoform levels. Pathologically, when 3R and 4R tau isoforms are not at equal ratios, tau aggregation takes place; however, the underlying mechanism remains unclear [8,9,10]. 

Oxidative stress and chronic inflammation have been shown to be the two key player at the root of neurodegenerative disease including AD [11]. During the progression of AD, the brain is more susceptible to the oxidative stress than the other organs because of the higher metabolic rate in the neurons. Moreover, nucleic acids, proteins, and lipids in neurons can be simply oxidized and then boost SPs as well as NFTs formation [12,13]. So, it has become clear that increased oxidative stress causes tau protein hyperphosphorylation in the early phase of AD. This hyperphosphorylation decreases the binding affinity of tau protein, destabilizes microtubules resulting in the formation of NFTs [14]. In addition, tau aggregates are capable of triggering the production of reactive oxygen species (ROS) via the activation of NADPH oxidase [14]. Hence, the development of antioxidants with approaches to decrease tau aggregation is considered as one of the most promising strategies for AD therapy [15]. Currently, Cannabidiol (CBD), a major constituent of *Cannabis sativa* L., gains considerable interest due to its antioxidant, anti-inflammatory, and neuroprotective beneficial properties [16]. In CBD, unpaired electrons are mainly distributed on ether and alkyl moieties making this natural substance remarkably antioxidant even more than alpha-tocopherol and vitamin C [17]. These groups in accompany with hydroxyl groups and double bonds contribute to increasing highest energy occupied (HOMO) value and reducing ionization potential (IP) value [17].

Several studies have shown that CBD might decrease AD initiation and progression [18,19]. For example, Vallee A et al. showed that CBD suppresses oxidative stress through activation of PPARγ (Peroxisome proliferator- activated receptor gamma) and inhibition of Aβ [18]. Moreover, Scuderi C et al. demonstrated that CBD induces ubiquitination of APP (Amyloid-beta precursor protein) in human neuroblastoma cells (SHSY5YAPP+) which leads to less Aβ production [19]. While potential neuroprotective properties of CBD have recently been appreciated, its mechanism of action as a tau aggregation inhibitor has not been fully explored. Therefore, we aim to investigate the effect of CBD on the aggregation of the recombinant human tau protein 1N/4R isoform using several biochemical and in silico methods. 

In this study, a human his-tagged tau protein was first generated in vitro using the pET-21a (+) expression vector in *Escherichia coli* BL21. Then, the kinetic diagram and binding affinity of heparin-induced tau protein aggregation were determined in the presence of different concentrations of CBD using different biophysical and biochemical analyses. In addition, the type of binding forces between CBD and tau protein was thermodynamically assessed. Finally, a deeper insight about the possible binding and mode of binding interaction of CBD with tau protein was simulated using molecular protein docking platforms. Taken together, this study provides new insights about the mechanism of action of a natural antioxidant substance, CBD, in altering tau protein aggregation which may offer a new avenue for the treatment of AD. 

## 2. Materials and Methods

### 2.1. Materials

The following products were used in this study: Thioflavin T (ThT) (Cat No. T3516, Sigma-Aldrich (Munich, Germany).), CBD (Cat No. C-045, Sigma-Aldrich (Munich, Germany).), and Heparin (Cat No. NC0521841, average MW = 15 KD, Celsus Laboratories (Cincinnati, OH, USA). SP Sepharose and Nickel Chelating Sepharose were obtained from Amersham Biosciences (Little Chalfont, England). All other chemicals were purchased from Merck (Darmstadt, Germany). 

### 2.2. Purification of Tau Protein

Human His-tagged tau 1N/4R cDNA was expressed using the pET-21a (+) expression vector in *Escherichia coli* BL21 strain DE3 (BL21(DE3) Singles™ Competent Cells-Novagen, Merk) as previously described in [20]. Briefly, bacteria were first grown at 37 °C in 50 mL Luria-Bertani (LB) broth supplemented with 100 μg/mL ampicillin at 180 rpm for 14 h. Then, cells were inoculated into 1 L LB broth for 4 h. When the OD of bacteria reached to the range of 0.6–0.8 at 600 nm, protein expression was triggered by adding β-D-1-thiogalactopyranoside (IPTG) (1 mM). After 4 h of incubation at 37 °C, cells were harvested and resuspended in lysis buffer (20 mM Tris-HCl, 0.2 mM MgCl_2_, 20 mM NaCl, 0.5 mM EDTA, 2 mM DTT, 0.1 mM PMSF and PH 7.5). Then, cells were disrupted using an ultrasonic disintegrator (Soniprep 150, MSE, London, UK) at 50% amplitude for 10 min on/off cycles on ice. After sonication, cell lysate was boiled for 30 min at 100 °C and centrifuged at 12,000× *g* for 30 min at 4 °C. Afterward, the filtered supernatants were loaded onto the purification column SP Sepharose. The column was rinsed 4 times with washing buffer containing 0.5 mM EDTA, 1 mM MgCl_2_, 50 mM NaCl, 20 mM Tris-HCl and PH 7.5 and proteins were eluted with elution buffer (0.5 mM EDTA, 1 mM MgCl_2_, 1 M NaCl, 20 mM Tris. Proteins). Proteins were further purified using the Ni-NTA column (Amersham BioSciences, Amersham, UK). After rinsing the columns with a 20 mM Imidazole, 20 mM Tris-HCl, PH 8 (4 times), Fractions of interest were collected in a buffer (6 M Imidazole, 20 mM Tris-HCl) [21]. The concentration of purified tau protein was measured using a UV-visible spectrophotometer (Varian bio-100) at OD 280 nm with excitation coefficient of tau protein 7700 M^−1^ cm^−1^ in the Beer-Lambert formula, and the purity was further assessed with SDS–PAGE. In addition, CBD absorbance was measured by UV-visible spectroscopy (Varian bio-100) to see whether it has any interference with tau protein absorbance or not.

### 2.3. Sodium Dodecyl Sulfate–Polyacrylamide Gel Electrophoresis (SDS–PAGE)

To semi-quantitatively check the purity of purified Tau protein, SDS-PAGE analysis was performed following [22]. Briefly, two volumes of (10 μL and 20 μL) tau protein (64 μM) were prepared in sodium dodecyl sulfate (SDS) sample buffer 5x (10 mL of stacking gel, 5 ml glycerol, 1 g SDS, 0.2 mL bromophenol blue and 1 mL 2-mercaptoethanol) separated on 12% SDS-PAGE gel. Then, tau proteins were visualized by Coomassie Brilliant Blue staining R250 (Bio Rad, Hercules, CA, USA) at MW of 59 kDa. 

### 2.4. Aggregation of Tau Proteins in the Presence of CBD

To assess the ability of tau protein polymerization in the presence of CBD, tau protein solution at final concentrations of 20 μM was incubated with different concentrations of CBD (0, 10, 20, and 40 μM) in 50 mM Tris-HCl (PH 7.5). DTT and heparin were also added to the samples at final concentrations of 5 mM and 5 µM respectively (Table 1). Samples were protected from light and were gently mixed at 180 rpm for 72 h at 37 °C. The aggregation rate of samples was measured using Thioflavin T assay [23].

### 2.5. Thioflavin T Assay

For exploring the kinetic of tau protein polymerization in the presence of heparin and different concentrations of CBD, a ThT-based fluorometric method was employed. First, 20 μL of samples were withdrawn and were incubated with 180 μL ThT for 10 min at 37 °C. Then, 20 μM tau and 10 μM ThT were used in this experiment. The assay was done using fluorescence spectrophotometer (Varian Eclipse) at excitation of 440 nm and emission of 455–600 nm, respectively. Excitation slit and emission slits were adjusted at 5 and 20 nm, respectively. The data were fitted against the sigmoidal curve using equation

(1) $$F=\frac{F_{max}}{1+\exp\;\left(-k_{app}\left(t-t_{1/2}\right)\right)\;}$$

where, F and $F_{max}$ are the fluorescence intensities at time t and at the end of the assay, respectively. In addition, $t_{1/2}$ is the required time for the formation of the half of the aggregation amounts and $K_{app}$ is the apparent rate constant [21,24]. 

### 2.6. Far-UV Circular Dichroism (CD) of Heparin-Induced Tau in the Presence of CBD

To investigate the changes in the secondary structure heparin-induced tau protein in the presence of CBD, far-UV CD spectra were recorded. At the end of the aggregation process, the samples (tau protein-induced with heparin in the presence of different concentrations of CBD) were diluted with the ratio 1:3 in Tris-HCl buffer PH 7.5 at 37 °C after 72 h. The evaluation was done in a 1 mm path length cuvette and a scan rate of 20 nm/min in the range of 190–260 nm, using Aviv model 215 Spectropolarimeter (Lakewood, NJ, USA). Each spectrum was scanned three times and subtracted from the buffer baseline. The average of the spectra is calculated and reported [22].

### 2.7. Far-UV CD of Native Tau Protein in the Presence of CBD

CD is a fast method for evaluating binding properties of proteins [25]. In this experiment, 20 μM tau protein was incubated with or without CBD at two different concentrations (2.3 μM and 14.96 μM) for 30 min at 37 °C. Then, the far-UV CD spectra were recorded. 

### 2.8. Fluorescence Quenching Assay

The structural changes of tau protein upon binding to CBD were documented using fluorescence spectroscopy. Briefly, to determine structural changes of tau protein upon binding to CBD, various concentrations of the quencher ranging from 0 to 14.9 μM CBD were added to the 20 μM tau protein (this concentration was constant until the end of the assay). The assay was accomplished by the fluorescence spectrophotometer (Carry eclipse) equipped with a Varian thermal controller at temperatures of 7 °C, 22 °C, and 37 °C and the Cuvette’s path length of 1 cm. The fluorescence spectra were documented in the range of 290–400 nm wavelength (with a slit width of 10 nm) after exciting at 275 nm (slit 5 nm). 

Quenching mechanisms may occur through either dynamic or static quenching or a combination of both processes. All these processes can be described by the Stern–Volmer equation. 

If the quenching is known to be dynamic, the Stern–Volmer equation will be as follows:

(2) $$\frac{F_{0}}{F}=1+K_{D}\left[Q\right]=1+k_{q}\tau_{0}\left[Q\right]$$

where $K_{D}$ is the Stern–Volmer quenching constant while the quenching is dynamic, $k_{q}$ is the bimolecular quenching constant rate, $\tau_{0}$ is the lifetime of the fluorophore in the absence of the quencher, and $\left[Q\right]$ is the concentration of the quencher (CBD) [26]. 

However, when the mechanism is static, the Stern-Volmer constant is calculated by the following equation:

(3) $$\frac{F_{0}}{F}=1+K_{S}\left[Q\right]$$

where $K_{S\;}$ is the Stern–Volmer quenching constant when the quenching is static. Finally, if a combination of both dynamic and static quenching is occurring, the Stern–Volmer equation will be as follows:

(4) $$\frac{F_{0}}{F}=\left(1+K_{D}\left[Q\right]\right)\left(1+K_{S}\left[Q\right]\right)$$

where this modified Stern–Volmer equation is a second-order polynomial in $\left[Q\right]$ and can also be expressed as:

(5) $$\frac{F_{0}}{F}=1+K_{app}\left[Q\right]$$

(6) $$K_{app}=\frac{\frac{F_{0}}{F}-1}{\left[Q\right]}=K_{S}+K_{D}+K_{S}K_{D}\left[Q\right].$$

After plotting $F_{0}/F$ versus [Q], an upward curvature was observed which attributes to the combined static and dynamic quenching. Then, the Stern–Volmer constants were calculated using the regression for the measured data points with the second-order polynomial of the Stern–Volmer equation (6).

To obtain the binding constants as well as the number of the binding sites of CBD on tau protein, Hill equation was used,

(7) $$\log\frac{F-F_{0}}{F}=\log k_{b}+n\log\left[L\right]$$

where $F_{0}$ and F are fluorescence intensities in the absence and presence of the quencher, respectively. In addition, $K_{b}$ is the binding constant, n is the number of the binding sites of CBD on tau protein, and $\left[L\right]$ is the concentration of the ligand (CBD) [27,28].

### 2.9. Thermodynamic Parameters of the Binding Assay

To detect which forces are involved in the binding assay, thermodynamic values were measured. Van’t Hoff equation was used to measure entropy $(∆S^{{}^{\circ}}$) as well as enthalpy ($∆H^{{}^{\circ}})$,

(8) $$\ln k=-\frac{∆H{}^{\circ}}{RT}+\frac{∆S{}^{\circ}}{R}$$

where R is the gas constant, T is the corresponding temperature, and k is the binding constant. Gibbs free energy was also calculated by Gibbs-Helmholtz equation to evaluate the spontaneity of the interaction) [29,30],

(9)  ∆G°=−RT lnk.

### 2.10. Stoichiometry of Tau-CBD Binding (Job’s Plot)

The stoichiometry of tau-CBD binding was measured using Job’s method [31,32]. In this assay, measurement of fluorescence change ($\Delta F=F_{protein}-F_{protein+ligand}$) was performed on a series of protein-ligand mixtures at room temperature, where the total molar concentration of CBD and tau was kept constant (20 μM) while their mole fractions were varied [33]. Maximum fluorescence intensity is normally reached at the composition corresponding to the stoichiometry of the predominant complex. 

### 2.11. Blind Docking of Tau-CBD

To obtain a better understanding of the interaction between CBD and tau protein, the best-fit docking pose configuration was constructed and analyzed using an online blind-docking tool available through Achilles server [34]. A PDB file for average structure of tau protein was first chosen from the ensemble of structures generated by Markus Zweckstetter group [10]. Further, the SDF file for CBD was downloaded from PubChem. The SDF file of CBD was then converted to the PDB format. Then, the blind docking was carried out by the Achilles platform and^.^ three best poses (binding mode) of the interaction of tau protein with CBD were simulated using this platform. Moreover, the 3D-view of the simulations was provided by Pymol [35]. In addition, the effect of CBD on the kinetic phase of heparin-induced tau protein was determined. 

### 2.12. Atomic Force Microscopy (AFM)

To prepare samples for AFM, 10 μL aliquots of samples (tau + heparin) and (tau + heparin + 40 µM CBD) were first gently mixed at 180 rpm for 100 h at 37 °C. Next, half of each sample was immediately placed on freshly cleaved mica and incubated for 5 min to be absorbed. Then, the mica surface was washed two times with filtered MiliQ water for 30 s and dried under a vacuum. Finally, images were taken using a non-contact mode AFM microscopy (AFM: Veeco AutoProbe CP- Research, tapping mode: 50 kHZ, oscillating frequency and spring constant: 15 N/m, tip radius: 10 nm) [21].

## 3. Results

### 3.1. In Vitro Generation of Human His-Tagged Tau Protein

**Figure 1:**
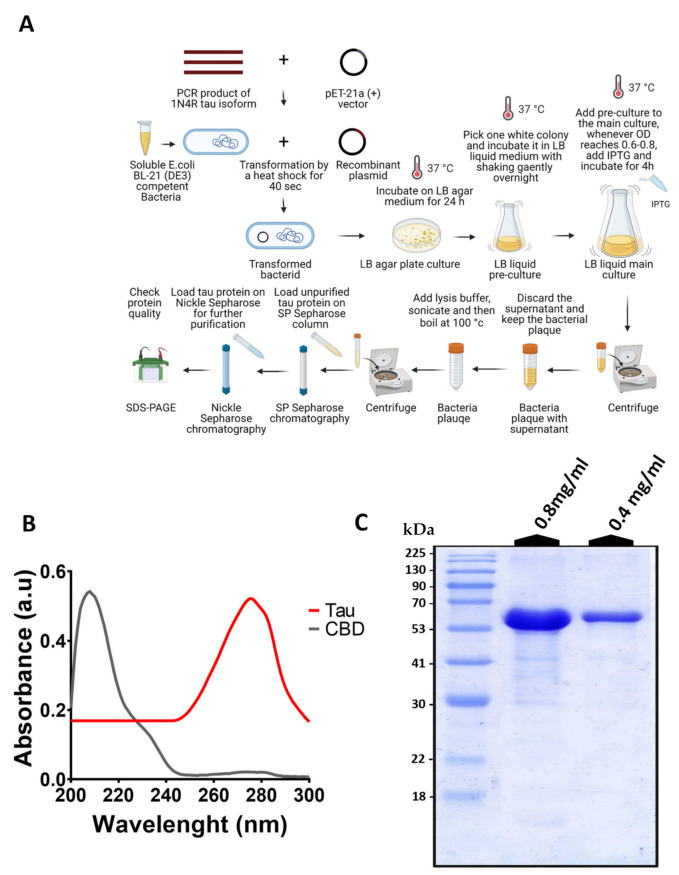
In vitro generation of human his-tagged tau protein. (**A**) Scheme of the main steps of his-tagged 1N4R tau protein production using the pET-21a (+) expression vector in *Escherichia coli* BL21 and purification by Ni-NTA and SP Sepharose columns. (**B**) The absorbance of extracted tau protein with a peak at 280 nm and the absorbance of CBD with a peak at 207 nm by UV-visible spectroscopy. It indicates that there is no interference between CBD and tau protein absorbance. (**C**) The quality of purified tau protein using SDS-PAGE. Representative SDS-PAGE of purified tau protein (~59 kDa) at two different concentrations of 0.8 mg/mL and 0.4 mg/mL. The same trends were detected in the SDS-PAGE data of at least three independent samples (*n* = 3).

In this study, to evaluate the impact of CBD on the aggregation of tau protein, human his-tagged tau protein was generated in vitro using the pET-21a (+) expression vector in *Escherichia coli* BL21 (Figure 1A). The absorbance of tau protein and CBD was assessed using UV-visible spectroscopy. One major peak around 280 nm for tau protein and one major peak around 207 nm for CBD were detected (Figure 1B). This result indicates that there is no overlap in the absorbance of CBD and tau protein (Figure 1B). Based on the Beer-Lambert law, A=εcl, where A is absorbance, ε is molar absorption coefficient, c is the concentration of tau protein, and l is the path length. Hence, we have 0.4905=7700×c×1, giving us the total tau protein concentration approximately equal to ~64 ± 3.84 μM. The quality and purity of extracted tau protein were then checked using SDS-PAGE. Based on the SDS-PAGE results and in consistent with the previous reports, the band corresponding to tau proteins was detected at MW around 59 kDa [21,22]. In addition, detecting only one band in the SDS-PAGE proved the purity of the tau protein fraction. 

### 3.2. Inhibitory Impact of CBD on the Formation of Amyloid Fibrils of Tau Protein

**Figure 2:**
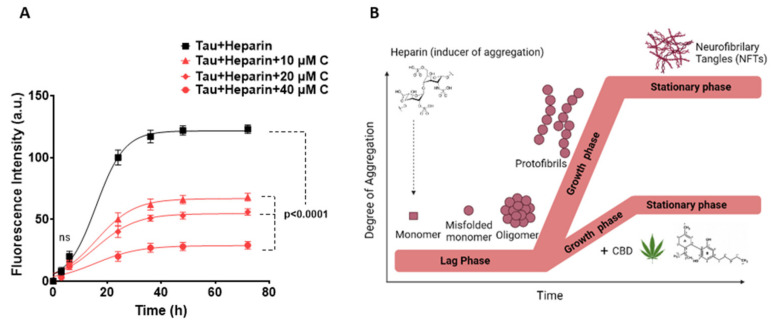
Aggregation kinetics of heparin-induced tau protein in the absence and presence of CBD. (**A**) ThT fluorescence assay to evaluate the kinetics of tau protein aggregation in the absence and presence of 10, 20 and 40 μM CBD after pre-induction with heparin. The aggregation kinetics of tau is plotted as fluorescence intensity of tau protein (a.u.) vs. time (h). The best-fitting curves for aggregation kinetics of tau follow a sigmoidal profile and a reduced rate appears in the presence of 10, 20, and 40 µM CBD. In addition, the kinetic changes of aggregation of tau alone were measured as zero constantly over time, so this result is not added in the kinetics plot. (**B**) Schematic representation of the three phases in the tau protein aggregation including lag phase, growth phase, and steady phase using the heparin inducer in the presence and absence of CBD. All data points are given as mean ± SD of three independent experiments (*n* = 3). *P*-value < 0.0001 is considered as statistically significant as determined by one-way ANOVA (Dunnet test) for the comparison between each data point vs. tau + heparin.

Aggregation of tau protein normally occurs through the nucleation-dependent fibril polymerization in three phases: activation and nucleation (Lag phase), elongation (growth phase), and steady phase (PHFs) (Figure 2B). In order to find out whether CBD is capable of inhibiting tau aggregate formation or not, a Thioflavin T assay was performed. In this assay, a small fluorescent molecule, namely Thioflavin T (ThT), strongly binds to amyloid fibrils which can be used for the quantification of amyloid fibrils. 

The kinetic diagram of heparin-induced tau protein aggregation in the presence of different concentrations of CBD was determined and fitted to the equation number (1). The heparin-induced tau protein aggregation happens in the sigmoid shape curve suggesting that the tau aggregation occurs in the above-mentioned three phases (Figure 2A, B). The steady phase became plateau nearly after 72 h, meaning that the aggregation did not proceed further. As shown in Figure 2A, by increasing the concentration of CBD, the fluorescence intensity was significantly reduced as compared to Tau + heparin (*p*-value < 0.0001). 

In addition, the results show that $F_{max\;}$ decreases significantly by increasing the CBD’s concentration up to 40 μM (Table 2), meaning that less tau aggregates are bound to ThT. Moreover, reduction in aggregation growth rate $K_{app\;}$ and enhancement in time, required for the formation of half amounts of tau aggregates while increasing CBD concentration, shows that CBD is lowering the aggregation rate (Table 2). Overall, these results confirm that CBD has an inhibitory effect on the amyloid fibrils formation during the tau protein aggregation. 

### 3.3. Structural Transformations of Heparin-Induced Tau Protein in the Presence of CBD

**Figure 3:**
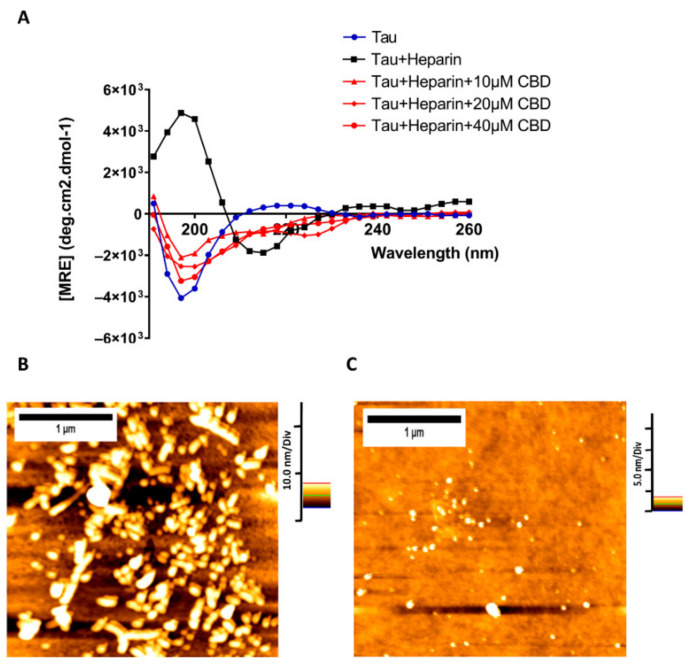
CBD is capable of reducing tau protein polymerization. (**A**) Far-UV CD spectra of 20 µM heparin-induced tau protein in the absence and presence of three concentrations of CBD (10, 20, and 40 µM) to monitor the changes in the secondary structures of tau aggregation after 72 h. Three independent experiments (*n* = 3) were performed to record the spectra. (**B**) AFM image of heparin-induced tau protein. (**C**) AFM image of heparin-induced tau protein in the presence of 40 µM CBD.

In order to obtain more insight about how CBD can affect the structural transformations of heparin-induced tau protein, Far-UV Circular Dichroism (CD) was used to assess the secondary structures of this protein. Tau protein has a random coil structure, when it aggregates it usually converts to a beta-sheet structure. For the random coil structure, the spectrum has a great negative band at approximately 204 nm and a small positive transition at 220 nm while the spectrum for beta-sheet protein structure has a negative band at 217 nm. As shown in Figure 3A, a random coil structure is related to the monomeric tau protein and a beta-sheet structure is more relevant to the aggregated form of tau protein. 

In the presence of 10 µM CBD, heparin-induced tau showed the same negative transition around 204 nm, with less negative molar ellipticity and a small transition at 220 nm demonstrating that CBD can change the beta coil structure of heparin-induced tau into a structure with few beta-sheets (Figure 3A). Increasing the concentration of CBD to 20 µM led the structure of heparin-induced tau to be similar to the random coil with more negative ellipticity at 204 nm with a small transition at 220 nm. Finally, in the presence of 40 µM CBD, heparin-induced tau has the most similar structure to the random coil but not exactly the random coil with the most negative ellipticity at 204 nm and a slight transition at 220 nm (Figure 3A). These results demonstrate that increasing CBD concentration can change the structure of tau protein more towards a structure close to the random coil.

AFM was further applied to investigate the morphological transformations of heparin-induced tau protein in the presence of 40 μM CBD. AFM images visually proved that twisted filaments are the result of tau protein aggregation induced by heparin (Figure 3B) while CBD causes inhibiting the formation of tau protein fibrils (Figure 3C). Taken all together, these results proved that CBD is capable of reducing tau protein polymerization.

### 3.4. Structural Transformation of Native Tau Protein in the Presence of CBD

**Figure 4:**
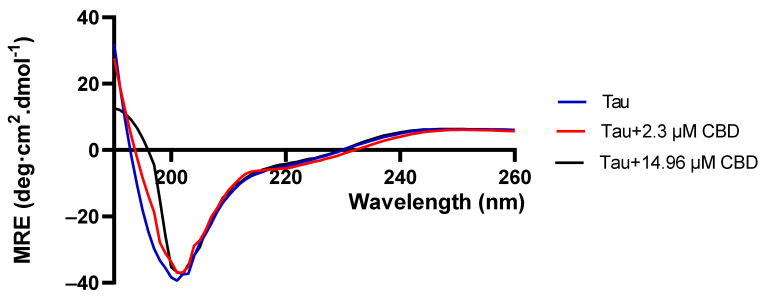
Far-Uv spectra in order to find out about CBD effect on tau protein structure. CBD transforms tau protein’s structure. Tau protein was incubated with CBD at concentrations of 2.3 µM and 14.96 µM for 30 min at 37 °C. Three independent experiments (n = 3) were performed to record the spectra.

In order to investigate the effect of CBD on the conformation of native tau protein in the absence of heparin, a Far-UV Circular Dichroism (CD) was employed. The selected concentrations of 2.3 μM and 14.96 μM for CBD were based on the quenching assay, where at 2.3 μM of CBD the binding sites of tau protein are not saturated but at 14.96 μM of CBD, tau protein’s binding sites become fully saturated. Hence, it concludes that CBD causes slight changes in the conformation of tau protein (in the CBD concentrations of 2.3 µM and 14.96 µM) (Figure 4) but it cannot promote tau aggregation. However, these small changes caused by CBD may alter the conformation of tau protein from the random coil to a rigid and structured conformation, so heparin will not be capable of tau protein aggregation as heparin induces tau protein aggregation when it has a random coil structure. 

### 3.5. CBD Binding to the Tyrosine Residues of Tau Protein

**Figure 5:**
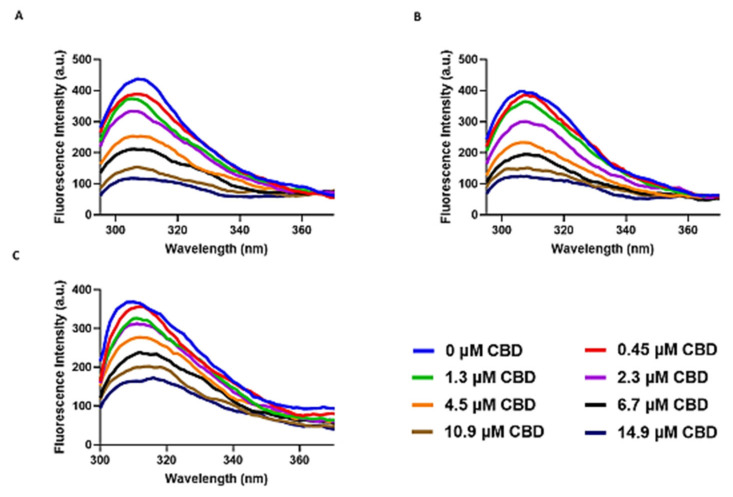
Intrinsic fluorescence quenching of tau protein by different concentrations of CBD ranging from 0–14.9 µM (0 µM, 0.45 µM, 1.3 µM, 2.3 µM, 4.5 µM, 6.7 µM, 10.9 µM, and 14.9 µM) in the Tris-HCl buffer and PH = 7.5. (**A**) at 7 °C (**B**) at 22 °C (**C**) at 37 °C.

We have applied an assay based on fluorescence signals that are intrinsic to the tau protein residues particularly Tyrosine (Tyr). In order to obtain insights into the fluorescence quenching mechanism, the intrinsic fluorescence of tau protein was measured in the presence of different concentrations of CBD ranging from 0–14.9 µM (0 µM, 0.45 µM, 1.3 µM, 2.3 µM, 4.5 µM, 6.7µM, 10.9 µM, and 14.9 µM) at 7 °C, 22 °C and 37 °C. Adding different concentrations of CBD to tau protein at 7 °C, 22 °C, and 37 °C caused quenching of only the fluorophore and Tyr in tau protein with a peak around 310 nm (Figure 5A–C). Increasing concentration of CBD more than 6.7 µM at temperatures of 7 °C, 22 °C, and 37 °C led the fluorescence intensity to become roughly stable, so it can be concluded that almost all the binding sites of CBD on tau protein were occupied and saturated.

These results also showed that CBD may interact with Tyr residue in tau protein and exert its quenching effect through this interaction. 

### 3.6. The Quenching Constants and Number of Binding Sites for CBD on Tau Protein

**Figure 6:**
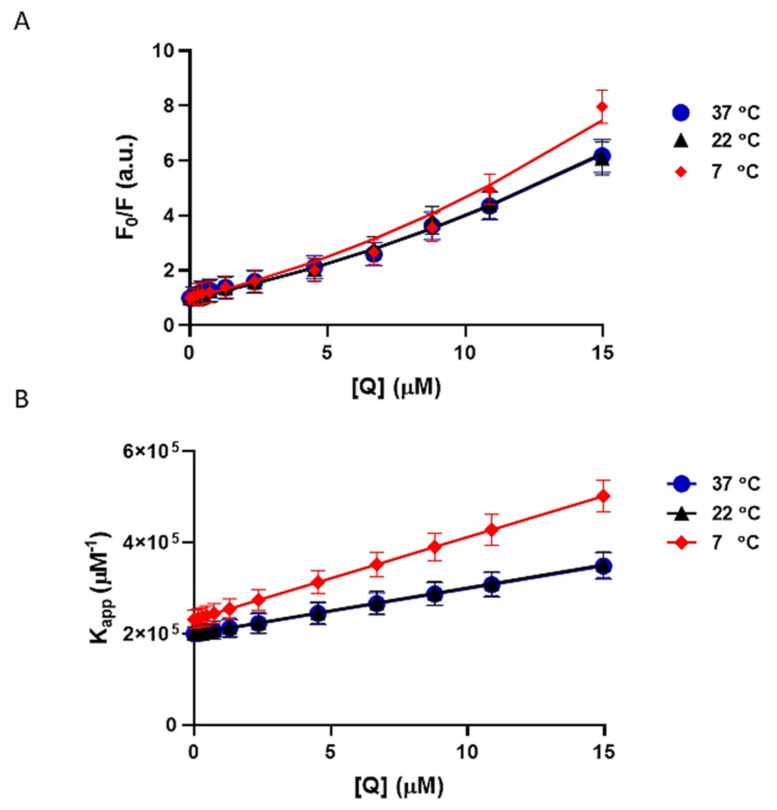
Modified Stern-Volmer plots of tau-CBD at 7 °C, 22 °C, and 37 °C. All data points are given as the mean ± SD of three independent experiments (*n* = 3). (**A**) Modified Stern–Volmer plot of tau protein fluorescence quenching at different concentrations of CBD from 0–14.9 µM (0 µM, 0.45 µM, 1.3 µM, 2.3 µM, 4.5 µM, 6.7 µM, 10.9 µM, and 14.9 µM) at 7 °C, 22 °C, and 37 °C. The data points are the results of the quenching experiment and the corresponding regressions are shown as lines. (**B**) The apparent quenching constant ($K_{app\;}$) plot of tau protein fluorescence quenching at different concentrations of CBD from 0–14.9 µM (0 µM, 0.45 µM, 1.3 µM, 2.3 µM, 4.5 µM, 6.7 µM, 10.9 µM, and 14.9 µM) at 7 °C, 22 °C, and 37 °C.

Fluorescence quenching usually takes place through either dynamic or static quenching or a combination of both processes. To figure out whether the quenching mechanism happens through dynamic or static or both, the intrinsic fluorescence of tau protein was measured at 7 °C, 22 °C, and 37 °C (Figure 5 and Figure 6). Here, plotting $F_{0}/F$ versus $\left[Q\right]$ reveals a slight positive deviation from linearity demonstrating that the tau-CBD quenching mechanism happens through both dynamic and static processes (Figure 6A). Positive deviations from linearity are usually observed when the order of quenching polynomial is higher than one. When the same fluorophore can be quenched both by collisional and complex formations, an upward curvature will be observed. Moreover, the Stern–Volmer constants ($K_{s\;}$ and $K_{q\;}$) were calculated using the regression for the collected data points with the second-order polynomial of the Stern–Volmer equation. The bimolecular quenching constant rate $K_{q\;}$indicates the efficiency of quenching. Larger values of diffusion-controlled limit $1\times 10^{10}\;M-1{\;S}^{-1}$ often demonstrate a binding interaction. Our results showed that $K_{q\;}$ was larger than the diffusion-controlled limit, validating a binding interaction. In addition, the plot of the apparent quenching constant $K_{app\;}$ versus $\left[Q\right]$ led to a straight line. For this mechanism, $K_{app\;}$ was calculated at each concentration of the quencher (Figure 6B). 

**Figure 7:**
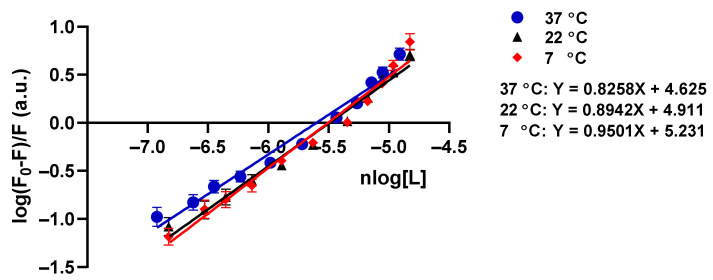
The Hill plots and intrinsic tau protein quenching constants of tau-CBD interaction at 7 °C, 22 °C, and 37 °C. Hill plot of tau protein fluorescence quenching at different concentrations of CBD from 0–14.9µM (0 µM, 0.45 µM, 1.3 µM, 2.3 µM, 4.5 µM, 6.7 µM, 10.9 µM, and 14.9 µM) at three different temperatures of 7 °C, 22 °C, and 37 °C. All data points are given as the mean ± SD of three independent experiments (*n* = 3).

The Hill equation (7) was further used to obtain the values of binding constants and binding sites (n) of CBD on tau protein. The Hill equation plots for three different temperatures are presented in Figure 7. Furthermore, the binding constants and stoichiometry number (n) values of the formed complexes in the temperatures 7 °C, 22 °C, and 37 °C are shown in Table 3. n ≈ 1 demonstrates that one CBD molecule may bind to tau protein, through the Tyr residues. Moreover, obtaining $K_{b\;}$ allows the measurement of thermodynamic parameters of the binding assay including standard entropy, enthalpy, Gibbs free energy changes, and major involved forces (Table 4). When the temperature is increased, it shows that n and Kb are decreased, but the reduction is not so significant due to the slight structural change of tau protein (Figure 5A–C and Table 3). In addition, the formation of the complex is an exothermic process due to the negative changes of $∆H^{{}^{\circ}}$. All the quenching parameters are shown in Table 3.

### 3.7. Type of Forces between CBD and Tau Protein

**Figure 8:**
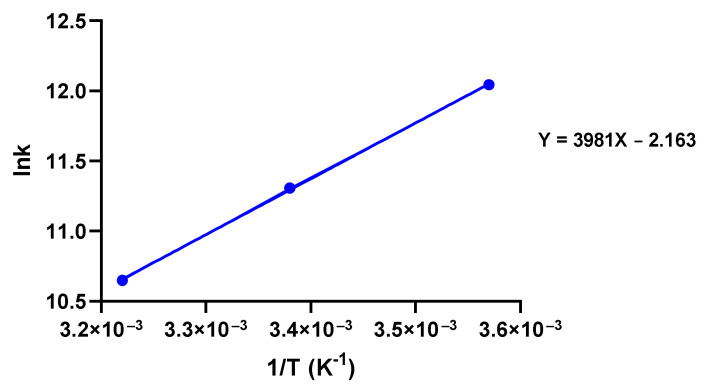
Van’t Hoff plot of fluorescence quenching and analysis of thermodynamic parameters to unravel the type of forces between CBD and tau protein.

In order to understand the forces involved in the interaction between CBD and tau protein, a thermodynamic analysis was performed. When small molecules bind to proteins, major forces are normally hydrogen bonds and Van der Waals, electrostatic, or hydrophobic. Based on former studies, the determination of the type of interaction between tau protein and CBD can be done by virtue of thermodynamic parameters, i.e. if ∆H>0 and ∆S>0 the interaction is hydrophobic, if ∆H<0 and ∆S>0 the interaction is electrostatic, and If ∆H<0 and ∆S<0 the interaction is due to hydrogen bonds and Van der Waals forces [36,37]. Van’t Hoff plot in Figure 8 shows that both enthalpy and entropy are negative, revealing that the interaction between tau and CBD is mostly through hydrogen bonds and Van der Waals forces. In addition, the small negative entropy shows that significant hydrophobic forces are present in tau-CBD interaction. Moreover, Gibbs free energy was calculated by equation (9) and its negative value means the interaction is spontaneous.

### 3.8. Detection of the Predominant Complex of Tau-CBD 

**Figure 9:**
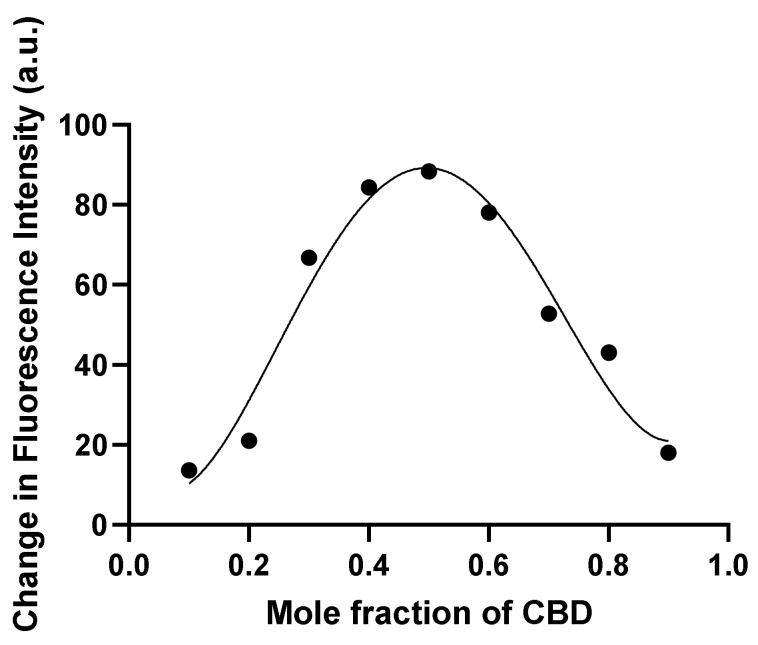
Stoichiometry determination of the predominant complex of tau-CBD. Changes of fluorescence intensity of a series of protein-ligand mixtures were measured. The total molar concentration of CBD and tau was constant (20 μM) but their mole fractions were varied.

Tau-CBD are capable of forming different complexes, since the $K_{D}$ values of the tau-CBD complexes are not equal. Therefore, Job’s method was applied in order to determine the predominant complex of tau-CBD (Figure 9). The total molar concentration of CBD and tau was constant (20 μM), while their mole fractions were varied. The maximum change in the fluorescence intensity was detected at the mole fraction of CBD $\chi_{X}=\frac{b}{b+a}=0.5$, where a and b are molecule number of tau protein and CBD, respectively and X represents CBD (Figure 9). Knowing that the mole fraction of CBD at the maximum fluorescence intensity is equal to the mole fraction of CBD in the predominant complex.

### 3.9. Best Cluster Selection in Docking Analysis

**Figure 10:**
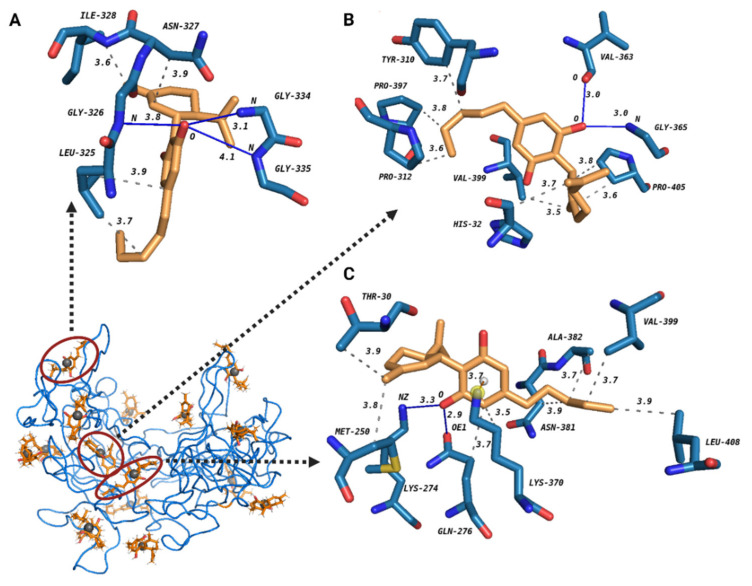
The 3D graphical image of three best tau-CBD docking complexes predicted by Achilles platform Tau protein and CBD are shown in blue and orange, respectively. The grey spheres are pseudo atoms. The best docking pose for three best-fitted clusters of tau-CBD interaction is presented in detail as complex A, complex B, and complex C. Hydrophobic interactions (in dash line), hydrogen bonds (in blue line) and one pi-cation interaction (in red line) as well as the distances between the donor and the acceptor are shown in all three tau-CBD complexes.

The Blind-docking was performed by the Achilles platform in order to gain further insight into the interaction between CBD and tau protein. Overall, this platform gave us information about forces and binding sites of CBD on tau protein. The Achilles platform constructed the best binding poses of 16 clusters for the interaction between CBD and tau protein. The 3D graphical image of three best tau-CBD complexes was then provided by Pymol (Figure 10). Two factors play an important role in the ranking of the best and the most effective complexes between tau and CBD in decreasing tau aggregation: (1) It is possible that CBD interacts with some important regions in tau protein such as hexapeptide motifs and heparin-binding sites, thus causing a reduction in tau aggregation; (2) Binding energy is another key factor that has a prominent role in choosing the best tau-CBD complex.

When a complex has lower binding energy, it means that the complex is more stable. Therefore, three best-fitted clusters in their best pose were selected based on one of the two factors explained above. Complex A suggests that CBD may have interfering (has a common binding site) interaction with four residues in heparin-binding sites: half of them through hydrophobic interactions (Ile328, Asn327), (Table S1) and the other half through hydrogen-bonds (Gly334, Gly335), (Table S2). In addition, we have to mention that the bond of Gly-335 with CBD is considered mostly electrostatic (weak hydrogen-bond) since the D-A distance is 4.08 $A^{{}^{\circ}}$ [38]. The values of binding energy for complex A, complex B, and complex C are −5.60 kcal/mol, −8.20 kcal/mol, and −6.10 kcal/mol, respectively. Therefore, complex B with the lowest binding energy is defined as the most stable tau-CBD complex. In addition, complex B has two common binding sites with heparin.

We speculate that CBD is capable of interacting with Gly365 and Val363 via hydrogen bonds (Table S4) which are located in heparin-binding sites. Furthermore, complex B demonstrates that tau protein may interact with Tyr310 (Table S3). These results are strongly in agreement with the quenching results. Complex C proposes that CBD possibly targets Gln276 through hydrogen bonds and hydrophobic interactions (Table S5 and Table S6) where it is located in the hexapeptide motif. Furthermore, CBD has two common binding sites with heparin (Asn381 and Ala382) (Table S5). In addition, CBD interacts with Lys370, which also is a site where heparin interacts with it, through cation-pi interaction (Table S7). These complexes have the most interfering sites among the others, showing that they are the most effective complexes in decreasing tau aggregation. Since the docking results showed that heparin and CBD may have common binding sites on tau protein, CBD inhibitory effects on tau aggregation may take place in the lag phase, inhibiting nucleation. Taken together, we can conclude that CBD is capable of mostly interacting and forming stable tau-CBD complexes with some key sites of tau protein, in particular hexapeptide motifs and heparin-binding sites, hence causing less tau aggregation formation. 

## 4. Discussion

Recently, several tau therapeutic strategies for the treatment of AD have been proposed which bring a new hope for the treatment of this life devastating disease [6,39]. Among them, hampering the tau aggregation has been, indeed, as one of the key strategies [40]. So far, a series of synthetic drugs have been tested for hampering tau protein aggregation; however, their side effects and toxicities discourage us from using them as a treatment [41]. On the other hand, bio-based products such as vitamins and herbal drugs show less toxicity and side effects [42]. Among many natural products, CBD has attracted much attention due to its antioxidant activity and anti-inflammation effect [16]. As it has been shown in the previous studies, inflammation is at the pathobiology root of ADs; therefore, applying the antioxidants has been highly appreciated for AD treatment [14].

Here, we have intensively studied the effect of CBD on tau protein aggregation in the presence of heparin as an inducer. Jangholi et al. showed that heparin, an aggregation promoter, induces aggregation by binding to hexapeptide motifs in R2 and R3 regions of tau protein, decreasing the electrostatic repulsion between interacting tau molecules [21]. For evaluating tau protein’s native structure in the presence of CBD, CD was performed. The results demonstrated that CBD does not cause tau protein aggregation and it may be capable of altering tau protein’s structure from a random coil to a rigid structure, decreasing tau protein’s tendency for aggregation. To explore the aggregation kinetics of heparin-induced tau protein in the presence of CBD, a ThT-based fluorometric method was then employed following equation (1). Furthermore, all measured kinetic parameters showed that in the presence of CBD less tau aggregates were bound to ThT. Thus, the results of ThT assay clearly proved that CBD has an inhibitory effect on tau aggregation. In order to gain more insight about the secondary structural transformation of heparin-induced tau in the presence of CBD, we employed an absorption spectroscopy method based on the differential of left and right circulatory polarized light, namely, CD. The secondary structure of tau protein before and after induction with heparin and in presence of different concentrations of CBD was studied. Ultimately, the CD records demonstrated that at the presence of 40 µM CBD, heparin-induced tau has the closest structure to the random coil with the highest negative ellipticity at 204 nm and a slight transition at 220 nm. This shows that CBD decreases tau aggregation and these results are in consistent with the ThT assay. Further, the morphology of tau protein aggregation was visualized in the presence and absence of CBD using AFM. Based on AFM images, CBD can inhibit tau protein aggregation. Overall, the AFM images were completely in line with CD and ThT results, proving that a lower number of amyloid fibrils was formed in the presence of CBD. 

In the quenching assay, a modified Stern–Volmer plot was used to measure quenching constants ($K_{s\;},K_{q\;}$) and to classify the mechanism of tau-CBD quenching [28]. Larger values of diffusion-controlled limit $1\times 10^{10}M-1S^{-1}\;$for $K_{q\;}$confirmed binding and high efficiency of quenching that was totally in line with our assumption about efficient quenching of tau-CBD on the grounds of CBD’s low ionization potential (IP), where IP value of CBD has been measured by R S. Borges et al. . The low IP value of CBD shows that it can easily undergo oxidation [17]. Moreover, it is widely accepted that the mechanism of quenching is an electron transfer from anion to fluorophore [28]. This suggests that quenching efficiency depends on the oxidation potential of the quencher [28]. As a result, tau-CBD quenching is a highly efficient quenching mechanism [17,28]. An upward curvature was observed in the modified Stern–Volmer plot which attributes to tau-CBD quenching that shows tau-CBD quenching happens through combined static and dynamic processes [28]. Furthermore, the binding constant ($K_{b\;}$) and the binding sites $\left(n\right)$ were measured by Hill plot. In addition, the type of interaction forces between CBD and tau protein was measured by Van’t Hoff plot by calculating enthalpy, entropy, and Gibbs free energy. By Van’t Hoff and Hill plots, we concluded that the interaction between tau protein and CBD is spontaneous and most likely via hydrogen and Van der Waals bonds as well as some non-negligible hydrophobic forces. 

In this study, beside the quenching assay, the computational approaches (ligand-protein docking) could precisely detect the possible interaction and binding sites of CBD on tau protein [17,26,28], while most of the studies in the literature have only performed the quenching assay to report the potential interaction forces and the number of binding sites of their proposed inhibitor of tau protein [22,43]. In particular, they have not determined the exact binding sites of their inhibitor of tau aggregation [22,43]. Furthermore, employing job’s plot showed that the predominant tau-CBD complex has the stoichiometry of 1:1 meaning that one molecule of CBD binds to one molecule of tau protein.

Finally, our data of ligand-protein docking further predicted the possible interaction and binding sites of CBD on tau protein. Ligand-protein docking can normally provide more insights about the interaction of tau-CBD, e.g. interfering (common binding) sites and certain important interactions. According to the previous studies, heparin has an important role either in the nucleation or elongation phase of tau protein aggregation [44,45]. Here, based on the ligand-protein docking analysis, we also speculated that the inhibitory effect of CBD occurs in the lag phase of heparin-induced tau due to the common binding sites of heparin and CBD, thus reducing tau aggregation by inhibition of nucleation phase. According to previous studies, CBD may target four important regions and reduce tau aggregation [21,46,47,48]. Moreover, it has been reported that almost each of the residues in the 275VQIINK280 hexapeptide motif is a potent driver of full-length tau fibril formation [46]. Therefore, CBD may target one of the important residues in this motif and decrease tau aggregation. In addition, it has been shown that if an inhibitor specifically interacts with Tyr310 in 306VQIVYK311 hexapeptide motif, it would be selected as a candidate for inhibition of tau protein aggregation [47]. Moreover, CBD may interact with the major heparin binding site of tau protein which is located in residues 327 to 391, causing reduction in tau aggregates formation. It has also been confirmed that electrostatic forces between positively charged residues of tau protein (such as Lys, Arg, and His) and heparin, a negatively charged inducer, have a critical role in tau-heparin interaction [49]. 

Our results showed that CBD targets Tyr 310 in one of the best fitted tau-CBD clusters and also Lys370 in another best fitted tau-CBD cluster. According to our results, CBD also targets multiple sites in the mentioned hexapeptide motifs (275VQIINK280 and 306VQIVYK311) as well as heparin-binding sites (residues 327–391). Here, by Addressing the stability of the tau-CBD complex and the exact possible binding sites of CBD on the key regions of tau protein, we firmly confirm that CBD is a powerful inhibitor of tau protein aggregation. 

## 5. Conclusions

Many studies have been so far examined the neuroprotective effects of CBD on several neurodegenerative diseases such as AD. However, to the best of our knowledge, the effect of CBD on tau protein aggregation, as one of the main causes of AD, is not well defined. Taken together, our study provides important steps to study the molecular effect of the CBD inhibitory action on the formation of amyloid fibrils of tau protein. Using the biochemical and in silico methods, we showed that one molecule of CBD may interact spontaneously with one molecule of tau protein and most likely through hydrogen Van der Waals bonds, and hydrophobic forces, therefore, inhibiting the lag phase of heparin induced-tau protein aggregation. In addition, CD results showed that tau protein tends to change its natural random coil structure to a rigid structure in the presence of CBD. Moreover, AFM proved less formation of tau aggregates in the presence of CBD. It should be noted that our conclusion is driven by the biochemical and in silico methods; therefore, further experiments are planned to study the impact of CBD in vivo. Our study suggests that CBD could have a tau therapeutic potency for the treatment of AD.

## Figures and Tables

**Table 1:** Tau protein aggregates preparation in the absence and presence of various concentrations of CBD.

Samples	Heparin (µM)	CBD (µM)	DTT (mM)	Tau (µM)
Tau	0	0	5	20
Tau + Heparin	5	0	5	20
Tau + Heparin + CBD	5	10	5	20
Tau + Heparin + CBD	5	20	5	20
Tau + Heparin + CBD	5	40	5	20

**Table 2:** Kinetic parameters of different CBD concentrations on tau protein aggregation collected from the best fit. By increasing CBD concentration, the kinetic values were significantly decreased. All data points are given as mean ± SD of three independent measurements (*n* = 3).

Samples	$F_{max}\;\left(a.u.\right)$	$k_{app\;}\left(h^{-1}\right)$	$t_{1/2\;}\left(h\right)$
Tau + Heparin	123.23 ± 3.40	0.19 ± 0.01	15.771 ± 0.82
Tau + Heparin + 10 µM CBD	68.45 ± 3.10	0.14 ± 0.01	16.3325 ± 0.81
Tau + Heparin + 20 µM CBD	56.11 ± 2.55	0.10 ± 0.02	17.0134 ± 1.10
Tau + Heparin + 40 µM CBD	29.78 ± 3.07	0.16 ± 0.01	17.8456 ± 1.07

**Table 3:** Intrinsic tau protein quenching constants of tau-CBD interaction at 7 ℃, 22 ℃ and 37 ℃ obtained from the modified Stern–Volmer equation (4) and Hill equation (7).

T (°C)	$K_{s}\times 10^{5}(M^{-1})$	$k_{q}\times 10^{12}\;(M^{-1}S^{-1})$	$K_{D}\times 10^{5}(M^{-1})$	$K_{b}\times 10^{4}(M^{-1})$	n
37	1.16 ± 0.05	5.80 ±0.23	1.16 ±0.05	4.21 ±0.12	0.82±0.01
22	1.00 ±0.03	5.03 ±0.15	1.00 ±0.03	8.14 ±0.32	0.89±0.02
7	0.99 ±0.03	4.99 ±0.10	9.99 ±0.03	17.01 ±0.51	0.95±0.02

**Table 4:** Thermodynamic parameters obtained by plotting Van’t Hoff, showing that both enthalpy and entropy are negative. It defines that there must be hydrogen bonds and Van der Waals forces. In addition, it shows that entropy is a small negative value due to the presence of significant hydrophobic forces.

$T\;\left({}^{\circ}C\right)$	$∆H^{0}\;\left(KJ\;mol^{-1}\right)$	$\;S^{0}\;\left(KJ\;mol^{-1}K^{-1}\right)$	$\;G^{0}\;\left(KJ\;mol^{-1}\right)$
37	−33.100	−0.018	−27.535
22	−33.100	−0.018	−27.805
7	−33.100	−0.018	−28.088

## Data Availability

Not applicable.

## Supplementary Materials

The following are available online at www.mdpi.com/article/10.3390/cells10123521/s1, Table S1: Hydrophobic interactions of cluster A. All distances are shown in $\left(Å\right)$. Table S2: Hydrogen-bond interactions of Cluster A. All distances are shown in $\left(Å\right)$. Table S3: Hydrophobic interactions of cluster B. All distances are shown in $\left(Å\right)$. Table S4: Hydrogen-bond interactions of Cluster B. All distances are shown in $\left(Å\right)$. Table S5: Hydrophobic interactions of cluster C. All distances are shown in $\left(Å\right)$. Table S6: Hydrogen-bond interactions of Cluster C. All distances are shown in $\left(Å\right)$. Table S7: Pi-cation interaction of Cluster C. All distances are shown in $\left(Å\right)$.
