# Supplementary material for: Cannabidiol Inhibits Tau Aggregation In Vitro
Source: Cells. 2021 Dec 13;10(12):3521. doi: 10.3390/cells10123521 (PMC8700709; doi:10.3390/cells10123521)
Supplement: Supplementary file 1 [file cells-10-03521-s001.zip › cells-1423915-supplementary.pdf]

Supplementary Materials

Table S1. Hydrophobic interactions of cluster A. All distances are shown in (Å).

Residue	AA	Distance D-A	Ligand Atom	Protein Atom
325A	LEU	3.69	19	2964
325A	LEU	3.94	4	2963
327A	ASN	3.88	12	2975
328A	ILE	3.55	16	2988

Table S2. Hydrogen-bond interactions of Cluster A. All distances are shown in (Å).

Residue	AA	Distance H-A	Distance D-A	Acceptor Atom	Donor Atom
326A	GLY	3.22	3.81	23	2966
334A	GLY	2.21	3.13	23	3042
335A	GLY	3.14	4.08	23	3047

Table S3. Hydrophobic interactions of cluster B. All distances are shown in (Å).

Residue	AA	Distance D-A	Ligand Atom	Protein atom
32A	HIS	3.73	14	357
310A	TYR	3.74	19	2824
312A	PRO	3.58	21	2850
397A	PRO	3.83	20	3661
399A	VAL	3.49	16	3677
405A	PRO	3.63	16	3723
405A	PRO	3.83	12	3724

Table S4. Hydrogen-bond interactions of Cluster B. All distances are shown in (Å).

Residue	AA	Distance H-A	Distance D-A	Acceptor Atom	Donor Atom
363A	VAL	2.24	3.00	3329	23
365A	GLY	2.13	3.02	23	3341

Table S5. Hydrophobic interactions of cluster C. All distances are shown in (Å).

Residue	AA	Distance D-A	Ligand Atom	Protein Atom
30A	THR	3.87	16	340
250A	MET	3.75	16	2263
267A	GLN	3.65	5	2506
370A	LYS	3.48	5	3386
381A	ASN	3.93	19	3508
382A	ALA	3.72	19	3519
399A	VAL	3.65	20	3677
408A	LEU	3.95	21	3762

Table S6. Hydrogen-bond interactions of Cluster C. All distances are shown in (Å).

Residue	AA	Distance H-A	Distance D-A	Acceptor Atom	Donor Atom
274A	LYS	2.78	3.29	22	2488
276A	GLN	2.46	2.93	2509	22

Table S7. Pi-cation interaction of Cluster C. All distances are shown in (Å).

Residue	AA	Distance D-A	Ligand Group	Ligand Atoms
370A	LYS	3.68	Aromatic	1,2,3,4,5,6
